# Characterization of *Streptococcus equi* subsp. *zooepidemicus* isolates containing *lnuB* gene responsible for the L phenotype

**DOI:** 10.1371/journal.pone.0284869

**Published:** 2023-04-28

**Authors:** María F. Azpiroz, Noelia Burger, Margarita Mazza, Grisel Rodríguez, Teresa Camou, Gabriela García Gabarrot

**Affiliations:** 1 Facultad de Ciencias, Fisiología y Genética Bacterianas, UdelaR, Montevideo, Uruguay; 2 CAMEC IAMPP, Colonia, Uruguay; 3 Laboratorio del Hospital de Colonia, Colonia, Uruguay; 4 CAMS IAMPP, Soriano, Uruguay; 5 Departamento de Laboratorios de Salud Pública, Ministerio de Salud Pública, Montevideo, Uruguay; Universidade de Lisboa Faculdade de Medicina, PORTUGAL

## Abstract

Within the framework of the β-hemolytic streptococci surveillance carried out by the National Reference Laboratory from Uruguay, three putative *Streptococcus equi* subsp. *zooepidemicus* (SEZ) were received from different health centers. Being these the first reports associated with human infections in Uruguay, the objective of this work was to confirm their identification, to determine their genetic relationship and to study their antibiotic susceptibility. Using four different methods, they were identified as SEZ, a subspecies which has been described as the etiologic agent of rare and severe zoonosis in a few cases in other countries. The three isolates presented different pulsotypes by PFGE; however, two of them appeared to be related and were confirmed as ST431 by MLST, while the remaining isolate displayed ST72. Their resistance profile exhibited an unexpected feature: despite all of them were susceptible to macrolides, they showed different levels of resistance to clindamycin, i.e. they had the so-called “L phenotype”. This rare trait is known to be due to a nucleotidyl-transferase, encoded by genes of the *lnu* family. Although this phenotype was previously described in a few SEZ isolates, its genetic basis has not been studied yet. This was now analyzed by PCR in the three isolates and they were found to contain a *lnuB* gene. The *lnuB* sequence was identical among the three isolates and with many *lnuB* sequences deposited in data banks. In conclusion, for the first time in Uruguay, three SEZ isolates recovered from non-epidemiologically related cases of human invasive infection were identified. Moreover, this is the first report about the presence of a *lnu* gene in the *S*. *equi* species, revealing the active lateral spread of the *lnuB* in a new streptococcal host.

## Introduction

*Streptococcus equi* subsp. *zooepidemicus* (SEZ) belongs to the large-colony Lancefield group C streptococci along with other species, like as *Streptococcus dysgalactiae* subsp. *equisimilis*, *S*. *dysgalactiae* subsp. *dysgalactiae*, *S*. *equi* subsp. *equi* and *S*. *equi* subsp. *rominatarum*. SEZ is an opportunistic pathogen, frequently isolated from horses, causing mild to severe infections such as adenitis, endometritis, pneumonia, among others. It is also associated with disease in a wide range of other domestic animals, including mastitis in cattle, polyarthritis in sheep and respiratory infections in dogs and cats [1–3]. In the case of humans, infection by this organism is a zoonosis linked with animal contact or with the consumption of inadequately pasteurized dairy products [2,4,5]. Although rare, SEZ can cause serious infections in humans such as meningitis, pneumonia, endocarditis, septic arthritis, osteomyelitis, myositis and sepsis, even leading to death. In addition, post-streptococcal sequelae of glomerulonephritis, rheumatic fever or hearing loss persist in some patients recovered from the infection [6–9]. SEZ shares >98% DNA sequence homology with *Streptococcus equi* subsp. *equi* and >80% with *Streptococcus pyogenes* [1,10]. Although it is considered an opportunistic pathogen, SEZ contains virulence factors homologous to those present in these two species such as the M-like protein SzP, superantigens SpeN, SpeP and SpeO, streptolysin S and the synthesis of a hyaluronic acid capsule in certain strains [11,12].

SEZ, like other members of group C streptococci, is typically susceptible to penicillin, which is considered the drug of choice to treat infections in humans as well as in horses and other animals. In addition to β-lactams, human isolates are commonly susceptible to other families of antibiotics such as glycopeptides, fluoroquinolones, daptomycin and linezolid. However, susceptibility to tetracyclines, macrolides, clindamycin, rifamycins and sulfonamides is highly variable among different isolates [2,13–16]. In the case of tetracycline, one of the strategies responsible for this antibiotic resistance is the specific ribosomal protection, whose most common enzymes are encoded by *tetM* and *tetO* genes [17]. Regarding clindamycin, the most common phenotype in streptococci includes, in addition to resistance to lincosamides, resistance to macrolides and streptogramin B (MLS_B_ phenotype) [2,18]. Less frequently, some isolates present a specific lincosamide resistance, called L phenotype, characterized by the susceptibility to macrolides. This rare phenotype is due to a nucleotidyl-transferase enzyme that modifies the antibiotic by adenylation. This function is coded by members of the *lnu* family, which comprises six different genes, *lnuA*, *B*, *C*, *D* and *E*, present in gram positive bacteria, and *lnuF* found in gram negative [19–21]. Among the first five, *lnuB* is the most broadly distributed, present in *Enterococcus faecium* and *E*. *faecalis* [22,23], *Staphylococcus aureus* [20,24,25] and *Streptococcus suis* [26], *S*. *agalactiae* [27] and *S*. *pyogenes* [28,29]; it was found associated to the *lsa(E)* multiresistance gene cluster responsible for the resistance to pleuromutilin, lincosamide and streptogramin A in all of these species expect for *S*. *pyogenes*, where *lnuB* was embedded in a different genetic environment. Particularly in SEZ, among reports that include the antibiotic resistance profile, four described isolates were resistant to clindamycin and susceptible to erythromycin, i.e. these would present the L phenotype [5,7,8,15]. However, in no case the mechanism responsible for this phenotype was analyzed.

Since 2006, passive surveillance of β-hemolytic streptococcal isolates has been carried out at the National Reference Laboratory (NRL), Uruguayan Ministry of Public Health. Between 2006 and 2021, 683 β-hemolytic streptococcal isolates, including 59 group C streptococci (30 invasive, i.e. isolated from normally sterile sites), were received from public and private laboratories throughout the country for further characterization. Among this group of isolates, three were identified as SEZ, being the first reports associated with human infections in Uruguay. Thus, the objectives of this work were to confirm the identification of the three isolates by different methodologies and to determine if they corresponded to a single lineage. In addition, the antibiotic susceptibility was phenotypically and genotypically studied.

## Materials and methods

### Bacterial isolates

Within the framework of the β-hemolytic streptococci surveillance, carried out by the National Reference Laboratory of the Uruguayan Ministry of Public Health, we regularly receive isolates followed by demographic information about the patients. Our routine protocol implies the assignment of a lab number to identify each isolate. After that, the patient information is anonymized and de-identified prior to analysis. This surveillance is approved by Dirección General de Salud, Ministerio de Salud Pública.

Between 2018 and 2019, three putative *Streptococcus equi* subsp. *zooepidemicus* (SEZ) from different health care providers were received at the NRL. Patients were elderly people between 75 and 90 years old and all of them had been in contact with domestic or farm animals. The first isolate (SEZ 559) was recovered from the cerebrospinal fluid of a patient who developed fatal meningitis. The second isolate (SEZ 567) was obtained from the blood of a patient who had fever of unknown origin. The third isolate (SEZ 594) was recovered from the blood of a patient with pneumonia. The last two patients had a good outcome. The food consumption habits of the three patients were unknown.

### Confirmation of the identification

Conventional biochemical tests (detection of Lancefield group C antigen, β-haemolysis activity, PYRase enzyme and bacitracin susceptibility), API 20Strep (bioMérieux, France), BBL-Crystal (Becton Dickinson Microbiology System, USA), Vitek2 (bioMérieux, France) and matrix assisted laser desorption/ionization time-of-flight (MALDI-TOF) MS (Bruker Daltonics) were performed.

### Antibiotic susceptibility

Antibiotic susceptibility was assessed using the disc diffusion method with discs of penicillin (10 units), erythromycin (15 μg), clindamycin (2 μg), tetracycline (30 μg), levofloxacin (5 μg), chloramphenicol (30 μg) and vancomycin (30 μg). For the first four antibiotics, the minimum inhibitory concentration (MIC) was determined using the E-test (bioMérieux, France). The protocols and the interpretation of the results were according to the guidelines of the Clinical and Laboratory Standards Institute (CLSI) [30].

### Detection of lincosamide and tetracycline resistance genes

A PCR approach was carried out to look for the *lnu* gene in the three isolates. First, the five *lnu* genes (*lnuA*, *B*, *C*, *D* and *E*) present in gram positive bacteria were searched for in data banks up to June, 2021. A representative sequence of the five *lnu* genes was chosen to conduct a BLAST search (https://blast.ncbi.nlm.nih.gov/Blast.cgi). These correspond to the following accession numbers: CP038184.1 for *lnuA*, CP031556.1 for *lnuB*, CP021772.1 for *lnuC*, CP033165.1 for *lnuD* and KF287643.1 for *lnuE*. No release of *Streptococcus equi* was found to contain the *lnu* sequences, or even traces of them. Taking into account that the *lnuB* gene is the most broadly distributed of *lnu* genes [19,26,27,29,31], the analysis was focused on this gene. Among streptococci, no matches were found in group C-Lancefield members, but six *S*. *agalactiae* strains resulted positive (ACs: CP031556, CP021773, CP021862, CP058666, CP059383 and KF772204). Considering that, among *lnuB* positive strains found in the public data banks, *S*. *agalactiae* was the most closely related species to SEZ, these six *lnuB* sequences were employed to design a *lnuB* primer pair. Specifically, forward and reverse primers were designed to amplify a DNA segment containing the entire 804 bp-gene, plus 16 bp upstream the start codon and ten bp downstream the stop codon (Table 1). *S*. *agalactiae* M6390 was taken as a reference of a *lnuB* positive strain (AC: HM209466) [31]. This strain was received at the NLR as part of the Latin American Program for Quality Assurance in Bacteriology and Antimicrobial Resistance (LA-EQAS), coordinated by the Pan American Health Organization and the Instituto Nacional de Enfermedades Infecciosas-ANLIS “Dr. Carlos G. Malbrán”. In the case of tetracycline resistance, the presence of *tetM* and *tetO* determinants were assayed by PCR using oligonucleotides previously described (Table 1) [32].

**Table 1:** Primer pairs used to detect *lnuB*, *tetM and tetO*.

Primer name	Primer sequence (5´-3´) ^(a)^	Annealing temperature	product name and expected size
lnuB-F	ATGAAAGGGTGAAGAAATGTTA	47°C	*lnuB* (830 bp)
lnuB-R	CGTTACTCTCCTATTCACTAATGT		
tetM-2	GAACTCGAACAAGAGGAAAGC	60°C	*tetM* (740 bp)
tetM-3	ATGGAAGCCCAGAAAGGAT		
tetO-F	AACTTAGGCATTCTGGCTCAC	55°C	*tetO* (519 bp)
tetO-R	TCCCACTGTTCCATATCGTCA		

^(a)^ Start and stop codons were underlined.

To prepare templates for PCR reactions, genomic DNAs were extracted with the “Genomic DNA Purification kit” (Thermo Scientific, #K0512). PCR reactions were performed in a total volume of 30 μL containing 1X buffer (BIORON), 200 μM of each deoxynucleotide triphosphate (Fermentas), 300 nM of each primer (Macrogen), 2U of High Taq polymerase (BIORON) and template DNA (100 ng and 800 ng for *tet* and *lnuB* amplifications, respectively). The conditions used were: 2 min at 94°C, followed by 30 cycles of 94°C for 30 s, 30 s at the annealing temperature, 1 min at 72°C, and a final extension step at 72°C for 5 min. Amplicons were purified with the MiniElute purification kit (Qiagen, #28006) and sequenced at Macrogen Inc. (Seoul, Korea).

Nucleotide sequences of the *lnuB* gene harbored by SEZ 559, SEZ 567 and SEZ 594 strains have been deposited at GenBank under the accession numbers OP355463, OP355464, OP355465, respectively.

### Pulsed-field gel electrophoresis (PFGE)

For PFGE analysis each isolate was collected with a swab from a fresh plate of overnight growth and suspended in 2 mL of PIV buffer (1.0 M NaCl in 10 mM Tris-HCl, pH 8). Then, the absorbance was measured using 1 mL at 578 nm and adjusted between 2.0 and 3.0. The suspension was centrifuged at 2,500 rpm for 5 min and the pellet was resuspended in 2 mL of PIV. After that 200 μL of the suspension was transferred to an Eppendorf and incubated in a bath at 50°C for 30 min. Then, 200 μL of 1% PFGE agarose grade (Amresco) in the PIV buffer was added and mixture was poured into plug molds (Bio-Rad). After 30 min at 4°C, the plugs were removed and incubated for 6 h at 37°C in lysis solution (6 mM Tris-HCl, 1.0 M NaCl, 100 mM sodium EDTA, 0.5% Brij 58, 0.2% sodium deoxycholate, 0.5% sodium lauroyl sarcosine, 1 mg/mL lysozyme, 8-U/mL mutanolysin, pH 8), followed by an overnight incubation in a 50°C water bath in ESP solution (0.5 M EDTA, 1% sodium lauroyl sarcosine, 0.2 mg/mL proteinase K, pH 8.0). The plugs were washed six times with TE buffer (10 mM Tris-HCl, 0.1 mM EDTA, pH 7.6) for 10 min in a 50°C shaker water bath each time. Then, plugs were stored in a fresh TE buffer at 4°C. One third of a plug was incubated with 20 U of SmaI (BioLabs) in the enzyme restriction buffer for 2 h at 25°C, and dispensed into wells of a 1% pulsed-field grade agarose gel (Amresco). The chromosomal digests were separated by PFGE, the first block with a switch time of 5 to 15 s for 10 h, and a second block with a switch time of 15 to 30 s for 9 h, both at a 120° angle with a voltage gradient of 6 V/cm at 14°C (CHEF-DR III, BioRad). *S*. *equi* subsp. *zooepidemicus* ATCC43079 was used as a reference strain and *Streptococcus pneumoniae* R6 as a molecular marker. The gel was stained with GelRed (Olerup SSP) and photographed with UV light. First, a visual inspection of the pulsotypes was carried out following the Tenover et al. 1995 criteria [33], and then the gel was analyzed with the Gel Compar II 5.1 program (Applied Maths, Kortrijk, Belgium).

### Multilocus sequence typing (MLST)

Multilocus sequence typing (MLST) based on *arcC*, *nrdE*, *proS*, *spi*, *tdk*, *tpi*, and *yqiL* was performed on the three SEZ isolates, according to Webb et al. 2008 [34]. Sequences were obtained from Macrogen Inc. (Seoul, Korea). PubMLST *S*. *equi* subsp. *zooepidemicus* database (http://pubmlst.org/szooepidemicus) [35] was used to determine the sequence type (ST) of each isolate and the ST obtained were compared against the total database.

## Results

Identification of the three isolates as *S*. *equi* subsp. *zooepidemicus* was confirmed by four different methods (Table 2). API 20Strep, VITEK 2 and BBL-Crystal biochemical differentiation systems identified the three isolates as SEZ with high probability. MALDI-TOF MS showed the same result with a score between 2.28 and 2.42.

**Table 2:** Identification of *Streptococcus equi* subsp. *zooepidemicus* isolates.

Isolate	Agglutination with group serum specimens	API 20Strep *(%)	BBL-Crystal *(^a^)	Vitek 2 *(%)	MALDI-TOF (score)
559	Group C	0463607 (99.9)	3667351541 (.9994)	071454265757651 (99)	2.40
567	Group C	0463607 (99.9)	1667351542 (.9999)	055454741757651 (96)	2.42
594	Group C	4463607 (99.6)	1667351503 (.9998)	051454365757651 (99)	2.28

(*) Biochemical profile.

^(a)^ Confidence factor.

**Fig 1:**
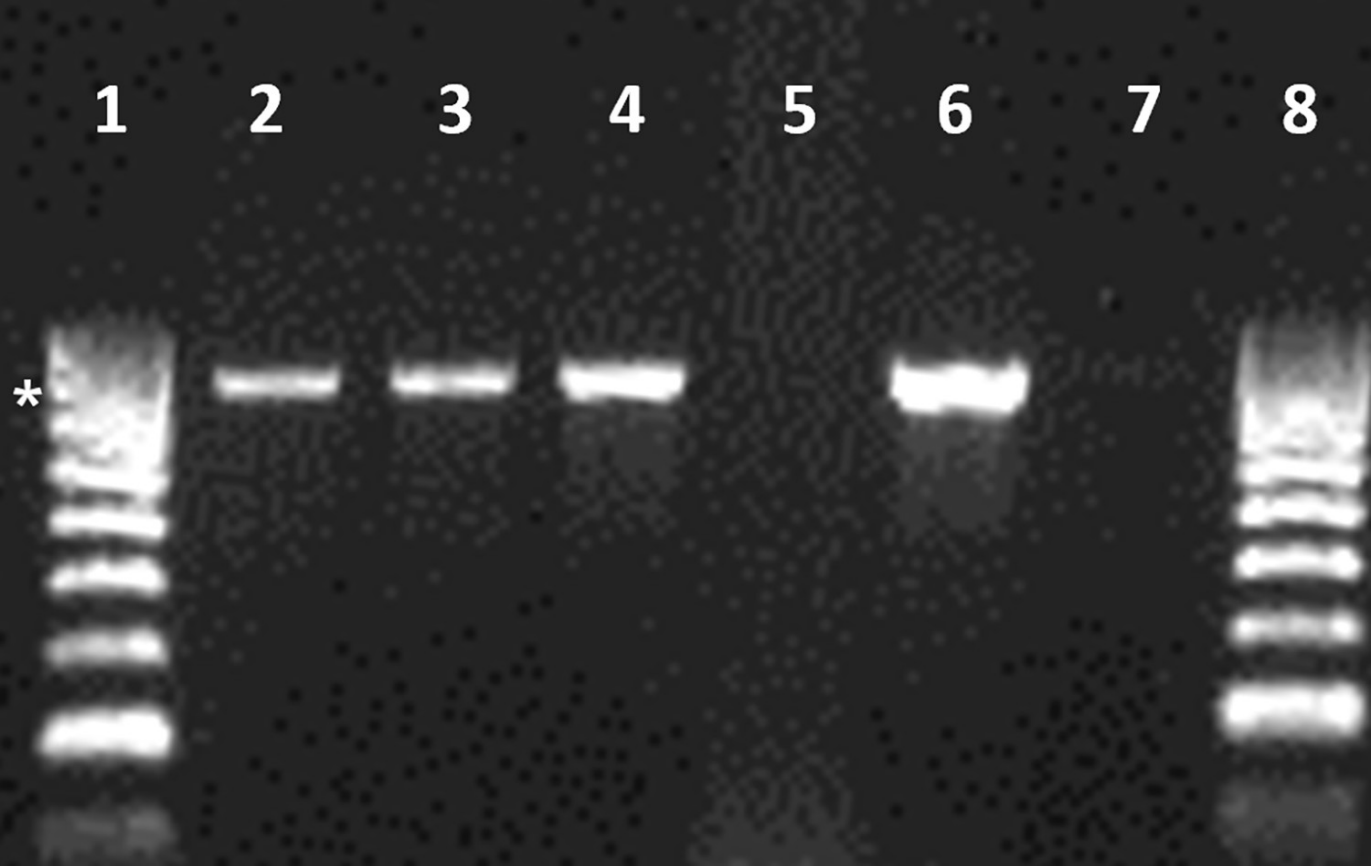
Amplification of the *lnuB* gene from the three SEZ isolates. Lanes: 1 and 8, 100 bp DNA HyperLadder (Bioline); 2, SEZ 559; 3, SEZ 567; 4, SEZ 594; 5, *S*. *equi* subsp. *zooepidemicus* ATCC43079 *lnuB*^*-*^ (AC: FM204884.1); 6, *S*. *agalactiae* M6390 *lnuB*^+^ (AC: HM209466); 7, No DNA. The 800 bp marker band is indicated with an asterisk (*).

Antibiotic susceptibility testing was assessed by the disc diffusion method and MIC determination by E-test. Isolates were susceptible to penicillin G, erythromycin, chloramphenicol, levofloxacin and vancomycin. Concerning tetracycline and clindamycin susceptibility, results differed among them. SEZ 559 and 594 exhibited intermediate resistance against tetracycline (MIC of 4 μg/mL both) while SEZ 567 was highly resistant (MIC of 32 μg/mL). PCR for the detection of *tetM* and *tetO* genes was performed on the three isolates. Only SEZ 567 was positive for *tetM* while in none of the isolates the *tetO* gene was amplified (S1 Fig). Analysis with clindamycin showed a borderline susceptibility by the disc diffusion method (20 mm and 19 mm, respectively) for SEZ 559 and SEZ 594 and a MIC of 0.50 μg/mL, interpreted as an intermediate resistance. SEZ 567, was categorized as resistant by both methods, although its MIC value was rather low (1 μg/mL). These results revealed that the three isolates presented the L phenotype, i.e. they were sensitive to erythromycin and resistant to clindamycin. Therefore, an analysis to identify the genetic basis of this phenotype was performed by PCR to detect the presence of the *lnuB* gene, as explained in Materials & Methods. An amplicon with the expected size (830 bp) was obtained from the three isolates (Fig 1). This positive result was achieved when the annealing temperature was reduced several degrees from that recommended by the enzyme producer (47° C instead of 52° C). Thus, an analysis of the conservation of the forward and reverse primers sequences was performed in data banks to hypothesize an explanation for this result. Indeed, while forward primer sequence was highly conserved among the *lnuB* releases, the region of the reverse primer appeared quite variable downstream the stop codon, which could explain the negative results under stringent conditions. On the contrary, for the reference strain *S*. *agalactiae* M6390, the PCR product invariably appeared as a strong band in all the conditions assayed. Amplicons from the three SEZ isolates were sequenced using primers lnuB-F and lnuB-R, and an identical 804 bp- *lnuB* sequence was found in all of them. It should be stressed that the first 6 bp and the final 14 bp of the gene are not corroborated since these sequences form part of the primers. The analysis in data banks revealed that the whole 804 bp sequence was the same as the *lnuB* one reported in many plasmids from *E*. *faecalis* and *E*. *faecium*, as well as in the chromosome of some *S*. *aureus* and *Streptococcus* spp., mainly *S*. *suis* and *S*. *agalactiae*. In addition, the same sequence recently appeared in a *S*. *dysgalactiae* DY107 genome release (AC: CP082206), i.e. from a species more closely related to *S*. *equi* than the remaining *lnuB* positive strains. Until now, no *lnuB* sequences have appeared in SEZ. Looking at the genetic environment of *lnuB* matches, this gene was found to be embedded in the *lsa(E)* multiple resistance gene cluster in most cases. In view of the susceptibility profile of our isolates, we particularly looked for a possible association between *lnuB* and tetracycline resistance genes. The analyses of data banks revealed that *tetM*, *tetO* and *tetL* (coding for one of the most common tetracycline efflux pump in gram positive clinical isolates [17]) are located in plasmids together with the *lnuB* determinant in some strains of *E*. *faecalis*, *E*. *faecium* and in one of *S*. *aureus* (AC: MK784778, MK465704, MN437484, MG957432, KY290886, HF586889, JX560992). In addition, a resistance gene cluster containing *tetL* was detected adjacent to the *lsa(E)* cluster in the chromosome of two *S*. *agalactiae* clinical isolates (AC: CP031556, MK102985), as well as the *tetO* gene in the chromosome of the *S*. *dysgalactiae* DY107 strain (AC: CP082206).

**Fig 2:**
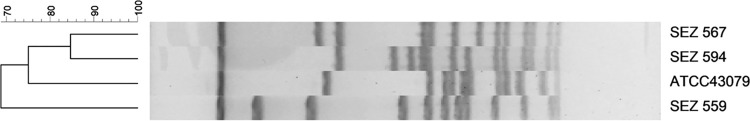
Pulsed-field gel electrophoresis (PFGE) and genetic relation analysis of SEZ isolates. The number of the respective isolate is indicated. *S*. *equi* subsp. *zooepidemicus* ATCC43079 was used as a reference strain. The gel was analyzed with the Gel Compar II 5.1 program (Applied Maths, Kortrijk, Belgium). Scale shows percentage of similarity.

Finally, the genetic relatedness of the three SEZ isolates was analyzed by PFGE and MLST. They all exhibited different pulsotypes, nevertheless, two of them, isolates SEZ 567 and SEZ 594, exhibited almost 85% of similarity in their profiles (Fig 2). Accordingly, SEZ 567 and SEZ 594 shared the same sequence type, ST431, while SEZ 559 belonged to ST72.

## Discussion

In this study, three isolates of *Streptococcus equi* subsp. z*ooepidemicus*–with no proven epidemiological link- were identified. This is the first report of human infections caused by this species in Uruguay. The isolates were recovered between 2018 and 2019 from elderly patients who developed invasive and severe infections (fatal meningitis, bacteremia, pneumonia). These findings correlate with previous observations pointing out SEZ as an uncommon but serious cause of human infections, particularly in elderly with underlying medical conditions or immunosuppression, and persons exposed to animals or animal products [3–5,36,37]. Group C streptococci meningitis is a rare type of infection, with approximately 50 cases reported, where SEZ appears as the most frequent causative agent [38–40]. Particularly in Latin-America, only three descriptions of meningitis caused by SEZ have been reported, one of them with a fatal outcome [40–42]. Thus, the meningitis caused by SEZ 559 is the fourth case identified in this region.

MLST and PFGE have been used in epidemiologic investigations of SEZ to determine the source of human infections, i.e. if the zoonosis resulted from direct contact with animals or from the consumption of dairy products [4,5,12,16,37,43–45]. PubMLST global database of *Streptococcus zooepidemicus* currently includes 830 SEZ isolates (http://pubmlst.org/szooepidemicus), most from Europe (77%). SEZ showed a wide genetic variation, i.e. in South America 23 isolates displayed 19 different ST and in North America 79 isolates displayed 55 different ST. Interestingly, two of our isolates share the same MLST, ST431. This ST was deposited in PubMLST database and corresponded to only one isolate from a horse infection in 2018 in Argentina (Bustos CP, personal communication). Taking into account that it was isolated in the same period of time and in the same region (our neighboring country, Argentina), an epidemiological relationship between this and our ST431 isolates could be suggested. Besides, SEZ 567 and SEZ 594 would be the first reports of human isolates with this sequence type. The other ST identified, ST72, was represented by five isolates in PubMLST, three were from human origin (UK and Brazil) and two from horses (UK and United Arab Emirates). We explored further the features of the Brazilian isolate because Brazil is also the other neighbor country to Uruguay. The above mentioned isolate was associated to a large outbreak of nephritis during 1997 and 1998, caused by consumption of unpasteurized cheese. The isolates were analyzed by PFGE and later one of them was assigned to ST72 after its genome was completely sequenced [4,11].

Regarding antibiotic susceptibility, a comparative analysis with previous published descriptions of human SEZ isolates that included antibiotic resistance profiles (around 20 reports) was carried out. Our results agree with previous descriptions: isolates were sensitive to penicillin G, erythromycin, chloramphenicol, levofloxacin and vancomycin [5,7,13,40–42,46]. As for tetracycline and clindamycin susceptibility, seven reports account for the resistance to one or both of these antibiotics [4,5,7,8,15,16,43]. Although data are very scarce, resistance to these two antibiotics tended to appear together in previous descriptions as it is found in our isolates [5,7]. Another important aspect to be mentioned is the low MIC values for clindamycin exhibited by the three isolates, i.e. two categorized as intermediate and one as resistant. As it was described before, MICs produced by the L phenotype are borderline or even below the breakpoints of resistance to clindamycin in streptococci and staphylococci strains [19,20,27,47–50]. Indeed, the four studies of SEZ that included the MIC for clindamycin reported values of 0.50, 1, 1.5 and 4 μg/mL [4,7,15,49].

Clindamycin is recommended for use in combination with penicillin as therapy for invasive *S*. *pyogenes* infections due to its inhibition on toxin production [51]; the same treatment has been applied for some infections caused by SEZ [52,53]. Thus, our report of *lnuB* in *S*. *equi* together with similar reports on other species of streptococci, stress the importance of continuous surveillance of clindamycin susceptibility.

In this work, the genetic basis for clindamycin and tetracycline resistance phenotypes was analyzed for the three SEZ isolates. The *lnuB* gene was found in all of them, which could explain the clindamycin resistance phenotype. To the best of our knowledge, this would be the first description of a *lnuB* gene in the context of *S*. *equi* subsp. *zooepidemicus*, and more generally the first time that a *lnu* determinant was found in the species *S*. *equi*.

It has been described that *lnuB* gene forms part of the *lsa(E)* multiresistance gene cluster, which basically comprises the resistance genes *aadE*, *spw*, *lsa(E)* and *lnuB*, always flanked by transposon sequences. It was postulated that this gene cluster has arisen in *Enterococcus* -that is harbored in plasmids and contains *erm(B)* and *aacA-aphD* in some cases- and has disseminated in staphylococci and streptococci by horizontal gene transfer [19,25,54]. As for streptococci, this multiresistance gene cluster is located in the chromosome of strains of some species of this genus [19,26,27,55], including the closely related *S*. *dysgalactiae* DY107 strain. In one of these descriptions, the *lsa(E)* cluster was found in an integrative and conjugative element of some *S*. *suis* isolates, which could be experimentally transferred by conjugation [26]. In the case of *S*. *pyogenes*, a clone containing a genomic island in which the *lnuB* is embedded in a different genetic environment was reported; i.e. *lnuB* is adjacent to *lsa(E)* gene and to the aminoglycoside-streptothricin resistance cluster [*ant*(6)Ia-*sat4-aph*(3’)III], but not to *aadE* and *spw* genes [28]. Concerning the possible association between *lnuB* and *tet* genes, some *lnuB*-*lsa(E)* positive strains of streptococci contained *tetM*, *tetO* and/or *tetL* in their chromosomes [19,26,55]. Particularly, *tet* determinants appeared in isolates from the two species most closely related to *S*. *equi*: *tetM* in the genome of an *emm*93.0 *S*. *pyogenes* strain and *tetO* in the chromosome of the *S*. *dysgalactiae* DY107 strain (AC: CP082206) [28]. Thus, the probable genetic linkage between *lnuB* and *tet* resistant genes should not be ruled out. In this sense, *tetM* was detected in one of the isolates, SEZ 567, which is in agreement with the MIC value reported for tetracycline. As to the remaining isolates, SEZ 559 and SEZ 594, their intermediate resistance against tetracycline suggests the possible presence of genes that encode efflux pumps such as *tetL*. Based on the above, we consider the possibility that the *lnuB* gene present in the three SEZ isolates could be located in an *lsa(E)* gene cluster near to a *tet* cluster, both of which might be part of a mobile genetic element acquired by horizontal gene transfer. More studies should be conducted to determine the genetic environment of the *lnuB* gene as well as its ability to be horizontally transferred from the SEZ isolates.

In conclusion, in a 16-year laboratory surveillance of streptococcal infections performed at the NRL, three independent SEZ infections were identified for the first time in Uruguay. The isolates presented the L phenotype and coherently harbored the *lnuB* gene. To the best of our knowledge, this is the first report of *lnu* gene in *S*. *equi* species, revealing active lateral spread of the *lnuB* gene, and most probably of the associated *lsa(E)* multiresistance platform, in a new bacterial host of the *Streptococcus* gender.

## Supporting information

S1 Fig Amplification to detect *tetM* and *tetO* genes in the isolates. Lanes: 1, No DNA; 2, *S*. *pyogenes tetM*^*+*^ strain (laboratory collection); 3, SEZ 559; 4, SEZ 567; 5, SEZ 594; 6, 100 bp DNA HyperLadder (Bioline); 7, No DNA; 8, *S*. *dysgalactiae* subsp. *equisimilis tetO*^*+*^ strain (laboratory collection); 9, SEZ 559; 10, SEZ 567; 11, SEZ 594. The 500 bp and 1000 bp marker bands are indicated with an asterisk (*).
(PPTX)

S1 Raw images (PDF)
