# Supplementary figures and images for: Characterization of Streptococcus equi subsp. zooepidemicus isolates containing lnuB gene responsible for the L phenotype
Source: PLoS One. 2023 Apr 28;18(4):e0284869. doi: 10.1371/journal.pone.0284869 (PMC10146458; doi:10.1371/journal.pone.0284869)

## Slide 1
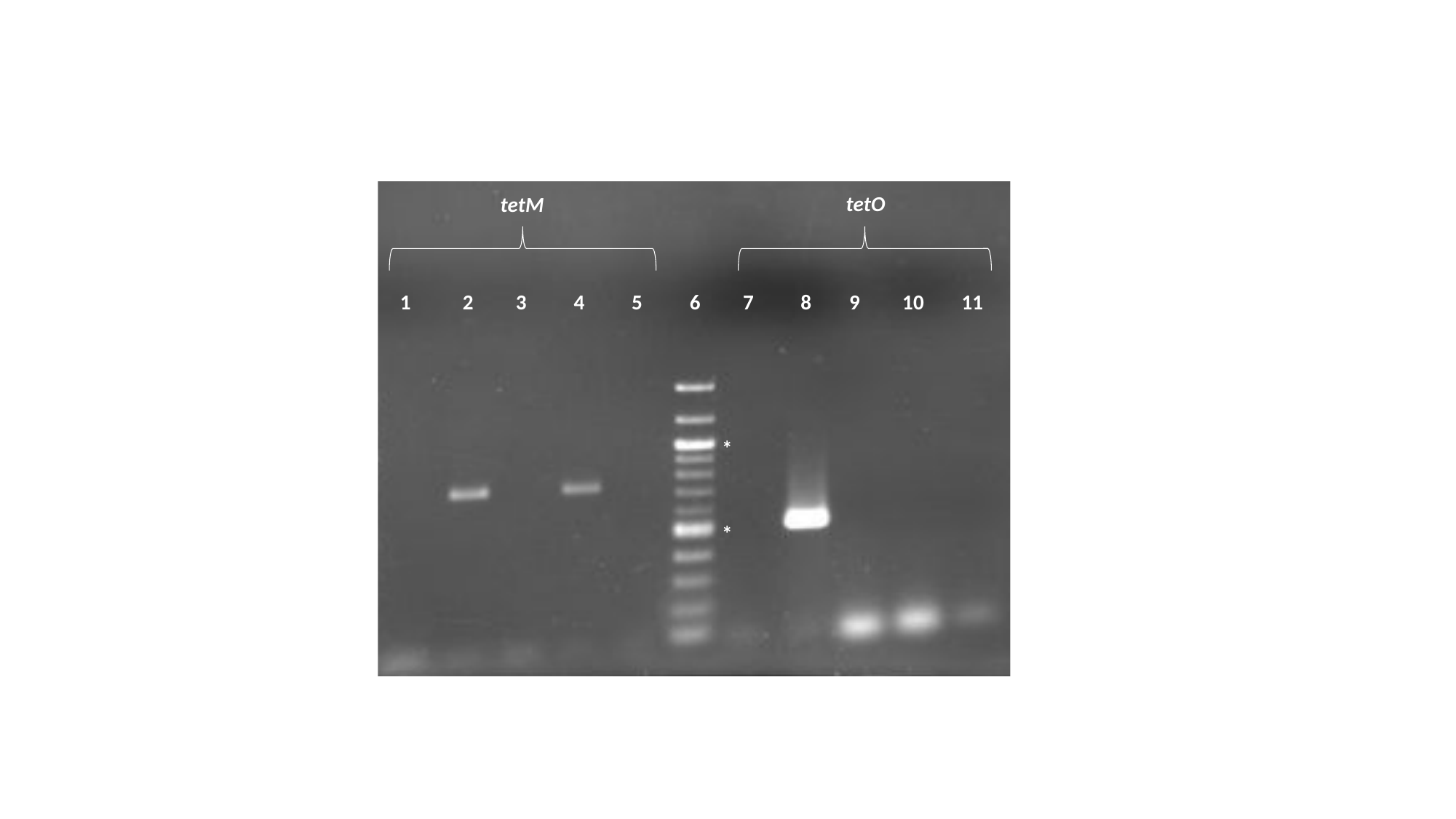

tetO
tetM
1 2 3 4 5 6 7 8 9 10 11
*
*

Supplement: S1 Fig — Lanes: 1, No DNA; 2, S. pyogenes tetM+ strain (laboratory collection); 3, SEZ 559; 4, SEZ 567; 5, SEZ 594; 6, 100 bp DNA HyperLadder (Bioline); 7, No DNA; 8, S. dysgalactiae subsp. equisimilis tetO+ strain (laboratory collection); 9, SEZ 559; 10, SEZ 567; 11, SEZ 594. The 500 bp and 1000 bp marker bands are indicated with an asterisk (*). (PPTX) [file pone.0284869.s001.pptx]
