# Supplementary material for: Characterization of Streptococcus equi subsp. zooepidemicus isolates containing lnuB gene responsible for the L phenotype
Source: PLoS One. 2023 Apr 28;18(4):e0284869. doi: 10.1371/journal.pone.0284869 (PMC10146458; doi:10.1371/journal.pone.0284869)

Figure 1
Capture with Bio-Rad Gel Doc XR+

1- Hipperladder (Bioline)
2- InuB from SEZ 559
3- InuB from SEZ 567
4- InuB from SEZ 594
5- InuB from SEZ ATCC43079
6- InuB from S. agalactiae M6390
7- InuB from no DNA
8- Hipperladder (Bioline)

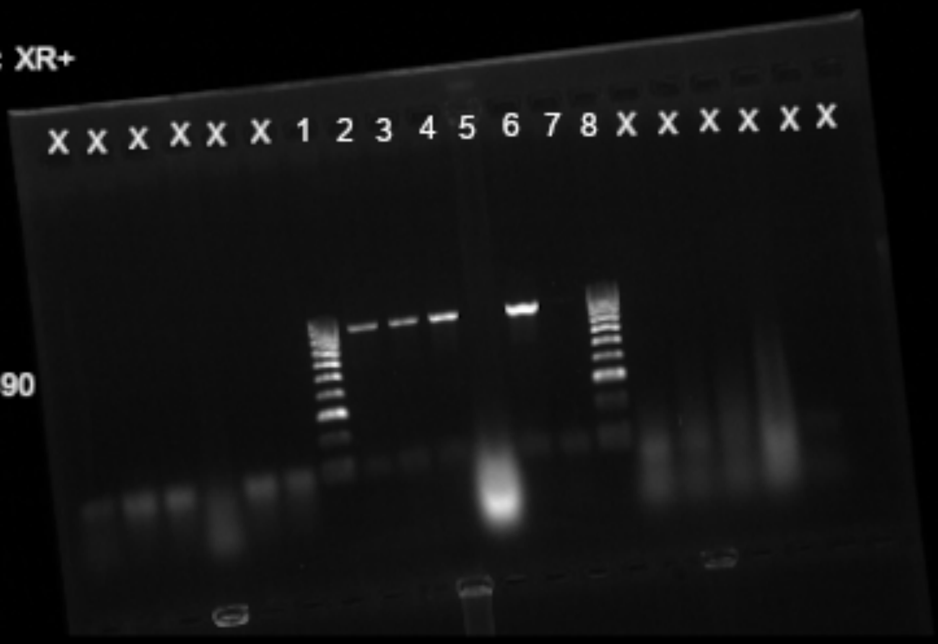

Figure 2
Capture with Bio-Rad Gel Doc XR+

1- PFGE S. prneumoniae R6
2- PFGE SEZ 559
3- PFGE SEZ 567
4- PFGE SEZ 594
5- PFGE SEZ ATCC43079
6- PFGE S. pneumoniae R6

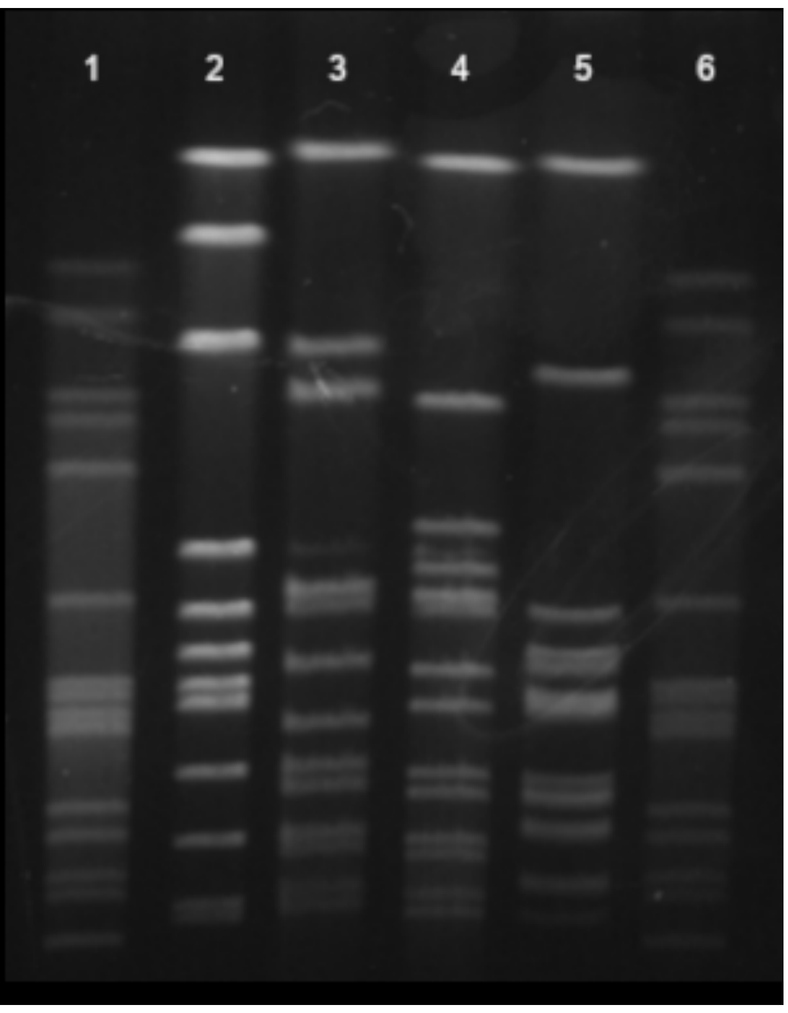

S1 Figure
Capture with Gel Logic 112 Camera
(Molecular Bioimaging, USA)

1- tetM from no DNA
2- tetM from S. pyogenes
3- tetM from SEZ 559
4- tetM from SEZ 567
5- tetM from SEZ 594
6- HyperLadder (Bioline)
7- tetO from no DNA
8- tetO from S. dysgalactiae subsp. equisimilis
9- tetO from SEZ 559
10- tetO from SEZ 567
11- tetO from SEZ 594

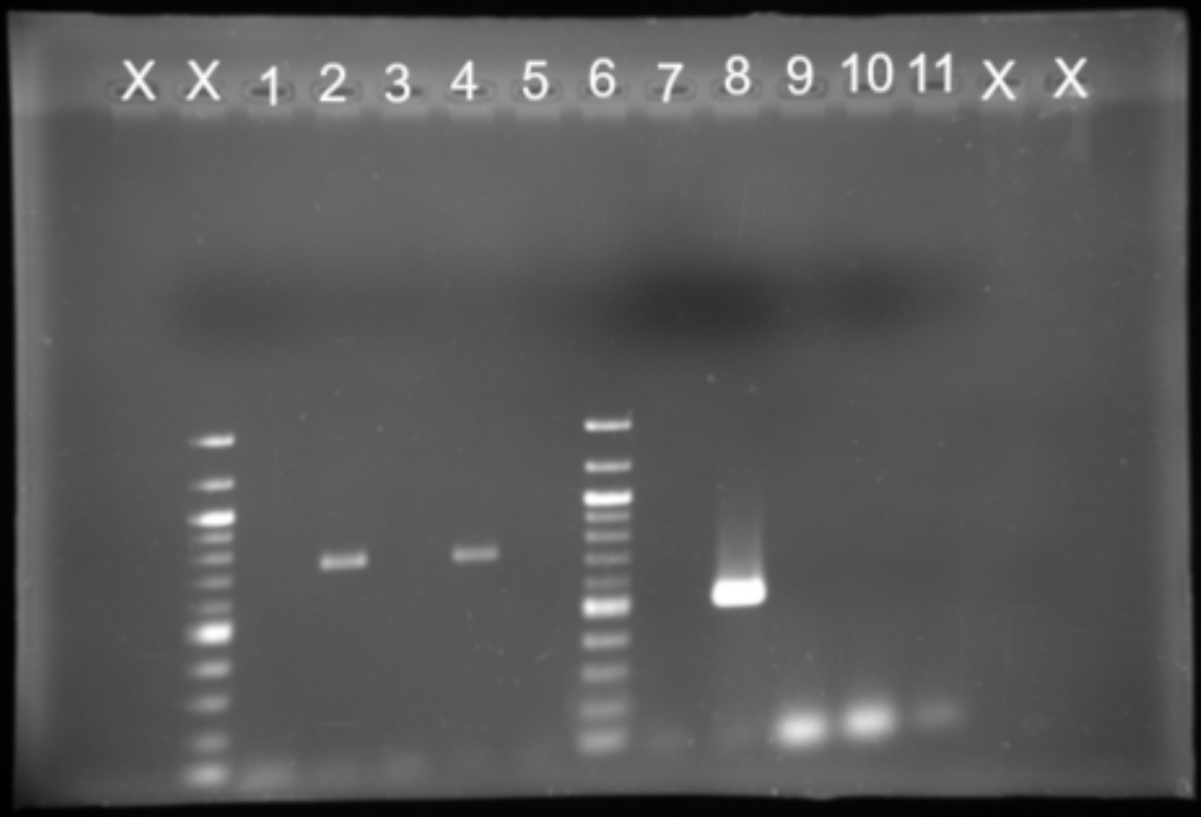

Supplement: S1 Raw images — (PDF) [file pone.0284869.s002.pdf]
